# Low LDL-C goal attainment in patients at very high cardiovascular risk due to lacking observance of the guidelines on dyslipidaemias

**DOI:** 10.1371/journal.pone.0272883

**Published:** 2023-05-22

**Authors:** Michal Vrablík, Ivana Šarkanová, Katarína Breciková, Petra Šedová, Martin Šatný, Aleš Tichopád

**Affiliations:** 1 3rd Department of Medicine, General University Hospital and 1st Faculty of Medicine, Charles University, Prague, Czech Republic; 2 CEEOR, Prague, Czech Republic; 3 Department of Internal Medicine and Cardiology, Faculty of Medicine, Masaryk University, University Hospital Brno, Brno, Czech Republic; 4 Department of Neurology, International Clinical Research Center, St. Anne’s University Hospital, Brno, Czech Republic; 5 Department of Neurology, Mayo Clinic, Rochester, MN, United States of America; 6 Department of Biomedical Technology, Czech Technical University in Prague, Prague, Czech Republic; University of Tampere, FINLAND

## Abstract

Dyslipidemias are defined as a wide range of abnormalities of the lipid profile. Treatment guidelines recommend aiming at lowering LDL-C. We investigated the adherence of Czech cardiologists to the dyslipidaemia treatment guidelines, especially in the management of patients with high and very high cardiovascular risk. In this retrospective cross-sectional multicentric study data from medical records of 450 adults with ASCVD, enrolled between June 2021 and January 2022, were analysed. Demographics, clinical outcomes, medical history, LLT treatment and other medications were collected. The physicians were to include patients at a very high risk of ASCVD and to complete a general questionnaire on their personal therapeutic preferences. Objectively assessed, only 80% of total patients (N = 450) enrolled in the study were at very high risk of ASCVD, and 12.7% of patients were at high risk of ASCVD, respectively. In total, 55 (13.1%) patients were diagnosed with familial hypercholesterolemia, and 39.1% of them had a positive family history of ASCVD. Generally, only 20.5% of patients reached the 2019 LDL-C goals– 19.4% of very high risk patients and 28.1% of high risk patients, respectively. 61% of the physicians preferred a slow and careful up-titration of the dose, which is contradictory to the guidelines. Only 17% of the physicians increased the statin dose or added/combined/changed the treatment to achieve the LDL-C goals as soon as possible. Surprisingly, in up to 61.5% of patients at very high risk who did not meet the LDL-C goals, their physicians stated subjective satisfaction with the treatment and considered no change needed. Among very high and high risk patients receiving lipid-lowering therapy, with high treatment adherence, the LDL-C goal attainment is very low and LLT utilization is rather sub-optimal. Improving observance of the guidelines by physicians bears a substantial potential for LDL-C goal attainment and thus improving overall benefit for patients for no additional costs.

## Introduction

Atherosclerotic cardiovascular disease (ASCVD) with its most common forms—coronary heart disease and stroke—remains the leading cause of mortality and premature death worldwide. Significant and causal contributors to the development and progression of ASCVD are lipid disorders–dyslipidemias [1].

Dyslipidemias are defined as a wide range of abnormalities of the lipid profile including increased plasma concentration of total cholesterol (≥ 5,2 mmol/L), LDL cholesterol (> 2.6 mmol/L) and triglycerides (> 1.7 mmol/L) that may be accompanied by low plasma concentration of HDL cholesterol (< 1.3 mmol/L for females or < 1.0 mmol/L for males) [2]. The primary causes of dyslipidemia are inborn errors of the lipoproteins metabolism, however an abnormal lipid profile can also be secondary to another uncontrolled disease or usage of specific drugs [3].

Several clinical studies have clearly demonstrated the causative role of increased plasma concentrations of apolipoprotein-B containing lipoproteins, mainly LDL in the process of atherosclerotic plaque formation [4–7]. The European Society of Cardiology (ESC) and European Atherosclerosis Society (EAS) have reflected these findings in the updated guidelines for the management of dyslipidaemias, proposing new stringent LDL-C goals together with a 50% reduction of LDL-C that should be achieved in high- and very-high-risk patients. Moreover, the combination of non-statin lipid-lowering therapy with statins was highly recommended, especially for these patient categories [8].

Two following international surveys, the EUROASPIRE V and DA VINCI showed a significant gap between the guideline recommendations and real clinical practice in the management of dyslipidaemias. Among patients receiving lipid-lowering therapy, less than 50% of high/very high risk patients achieved the LDL-C goals of the 2016 ESC/EAS Guidelines, with approximately 20% achieving the 2019 goals. Even though the guidelines recommend the combination of statin with non-statin LDL-lowering therapies to achieve the ambitious LDL-C goals, the usage of ezetimibe and PCSK9 inhibitors was low (9% and 1%, respectively) compared to statin monotherapy [9, 10].

These observations are in line with those from the Czech Republic. Despite the availability of the national adaptations of the 2016 as well as 2019 ESC/EAS Guidelines for the management of dyslipidaemias prepared by the Czech Society of Cardiology and the Czech Society for Atherosclerosis [11, 12], a significant proportion of patients still presents unsatisfactory control of risk factors along with a slow up-titration of the lipid-lowering drugs and a limited use of combinatory therapy [13]. The reason of the described situation is not entirely clear.

The objective of the presented retrospective cross-sectional study was to investigate the adherence of Czech cardiologists/internists to the dyslipidaemia treatment guidelines, especially in the management of patients at high and very high cardiovascular risk. The survey explored the physicians’ approaches and perceptions of their day-to-day practice with the aim to identify potential reasons of insufficient and delayed implementations of evidence-based recommendations into the real-world clinical practice.

## Methods

### Study design

A non-interventional cross-sectional multicentric study was performed in the Czech Republic. The study was designed, and the participants recruited in cooperation with the Czech Atherosclerosis Society, involving 46 specialists (internal medicine and cardiology) focusing on the treatment of patients at risk of ASCVD. Each physician should have included 10 consecutive very high risk patients. After completing all patient questionnaires, the physician also completed a general questionnaire that relates to their personal therapeutic preferences. 450 adults were enrolled between June 2021 and January 2022 (3 physicians included less than requested 10 patients). The patients were enrolled at routine clinic visits and anonymous patient data were collected from medical records using a standardized electronic report form (eCRF). The risk category stratification was performed according to the 2019 ESC/EAS Guidelines [8] by the research team after data collection.

The fully anonymized medical chart records of patients were analysed retrospectively, without intervention or participation of the patient. No treatment was studied as to its efficacy, effectiveness, or safety. There could therefore be no prejudice to the patient or his rights. The data contained no identifying information such as name and surname, initials, address of residence. The date of birth was limited to information about the year only and did not include information about a specific day or month. Given the high prevalence of ASCVD, the likelihood of identifying an individual based on the data obtained is extremely low. For this type of study, the Czech national law (Act No. 378/2007 Coll., on Pharmaceuticals) does not require ethics committee approval or patient´s consent. The study was registered and approved by the State Institute for Drug Control and published in its registry of non-interventional studies under the identification number 2108170000.

### Inclusion criteria

The patients had to meet the following inclusion criteria being in the very high cardiovascular risk; being aged ≥ 18 years and being prescribed LLT at least one year prior to the enrolment in the study. There were no exclusion criteria.

### Variables

The following data were collected for the patient questionnaire: demographics (weight, height, smoking status); blood pressure; lipid values; medical history; LLT treatment and other medications.

Statin intensity treatment was classified as a low-intensity statin therapy (daily dose lowers LDL-C by <30%; simvastatin 10 mg), moderate-intensity (daily dose lowers LDL-C by 30% to <50%; rosuvastatin 5–10 mg/day; atorvastatin 10–20 mg/day or simvastatin 20–40 mg/day) and high-intensity statin therapy (daily dose lowers LDL-C by ≥50%; atorvastatin 40–80 mg/day or rosuvastatin 20–40 mg/day) [8].

The treatment goals were evaluated by absolute level of LDL-C attained (<1.4 mmol/L; <1.8 mmol/L; <2.6 mmol/L and <3.0 mmol/L) for patients at very high risk, high risk, moderate and low risk, respectively [8].

In the general questionnaire, the following parameters were collected: criteria for the change in pharmacotherapy; attitude in case of insufficient response to the treatment; perception of the treatment effectiveness; objective parameters of LLT efficacy and goal of LLT.

### Statistical analysis

The data analysis was carried out using the SAS 9.4 software for Windows. The category variables were analysed by absolute and relative frequencies. All metric data were described by the mean and standard deviation.

## Results

### Patient characteristics

The physicians were asked to enrol patients at very high risk of ASCVD reflecting the risk stratification according to the 2019 ESC/EAS Guidelines [8]. Objectively, only 80% of total 450 patients enrolled in the study were at very high risk of ASCVD based on the guideline stratification, of whom 304 had a history of a cardiovascular event. 12.7% of the enrolled patients were at a high risk of ASCVD. We decided to include them into the analysis too despite the original aim of the study to analyse only very high risk patients. The rest of the patients at a moderate or low risk of ASCVD (5.8%), together with those with incorrectly entered values (1.3%) were excluded from the analysis. There were more male participants in the study– 64.4%. The average age of the males was 67.4±11.1, and the females 69.2±12.7. Obesity (BMI above 30 kg/m^2^) was present in 46.5% of the patients; 20.8% of the study subjects were active smokers. In total, 55 (13.1%) patients were diagnosed with familial hypercholesterolemia and 39.1% of the study cohort had a positive family history of ASCVD (Table 1).

**Table 1:** Patient characteristics.

Risk category	Very high + High	Very high	High
**Num. of patients**	418 (93.1%)	361 (80.2%)	57 (12.7%)
**Sex**			
***Female***	*149 (35*.*6%)*	*111 (30*.*7%)*	*38 (66*.*7%)*
***Male***	*269 (64*.*4%)*	*250 (69*.*3%)*	*19 (33*.*3%)*
**Age (years) mean (SD)**	68.1 (11.7)	68.1 (11.4)	67.8 (13.5)
**Female**	69.2 (12.7)	67.9 (13.2)	73.2 (10.0)
**Male**	67.4 (11.1)	68.2 (10.5)	57.4 (13.5)
**Systolic blood pressure (mmHg) mean (SD)**	135 (14.6)	135.4 (14.9)	131.87 (11.98)
**Diastolic blood pressure (mmHg) mean (SD)**	79.5 (9.2)	79.7 (9.0)	77.19 (10.0)
**BMI > 30 (obesity) kg/m** ^**2**^	195 (46.5%)	169 (46.8%)	26 (44.8%)
**Non-smoker**	331 (79.2%)	281 (77.8%)	50 (87.7%)
**Smoker**	87 (20.8%)	80 (22.2%)	7 (12.3%)
**Hypertension dg.**	342 (81.6%)	296 (82%)	46 (79.3%)
**Positive family history of ASCVD**	164 (39.1%)	150 (41.6%)	14 (24.1%)
**Type 1 diabetes**	5 (1.2%)	4 (1.1%)	1 (1.7%)
**Type 2 diabetes**	218 (52%)	180 (49.9%)	38 (65.5%)
**Chronic kidney disease dg.**	63 (15%)	58 (16.1%)	5 (8.6%)
**Familial hypercholesterolemia dg.**	55 (13.1%)	49 (13.6%)	6 (10.3%)
**Metabolic syndrome dg.**	148 (35.3%)	128 (35.5%)	20 (34.5%)
**Cardiovascular event(s)**	304 (72.2%)	304 (84.2%)	-
**Total Cholesterol (mmol/L) mean (SD)**	4.07 (1.19)	4.14 (0.82)	4.06 (1.24)
**LDL-C (mmol/L) mean (SD)**	2.21 (1.00)	2.18 (0.96)	2.36 (1.21)
**HDL-C (mmol/L) mean (SD)**	1.24 (0.33)	1.29 (0.32)	1.23 (0.33)
**ApoB (mmol/L) mean (SD)**	0.83 (0.26)	1.03 (0.44)	0.81 (0.24)
**TAG (mmol/L) mean (SD)**	1.69 (0.95)	1.67 (1.03)	1.69 (0.94)

ASCVD—atherosclerotic cardiovascular disease; BMI—body mass index; SD—standard deviation.

### Achievement of the LDL-C goals defined by the 2019 European Society of Cardiology/European Atherosclerosis Society Guideline

The mean value of LDL-C concentrations in patients at very high risk of ASCVD was 2.18 mmol/L, in high risk patients it was 2.36 mmol/L. The time between the follow-up cholesterol checks less than 12 months (as recommended) was performed in 85% of the patients. In both monitored groups, there was a decrease in the average value of LDL-C compared to the previous measurement; the mean value was 0.28 mmol/L, which represented 11% decline for very high risk patients, respectively 0.32 mmol/L with 12% decline for high risk patients (S1 Table).

**Fig 1:**
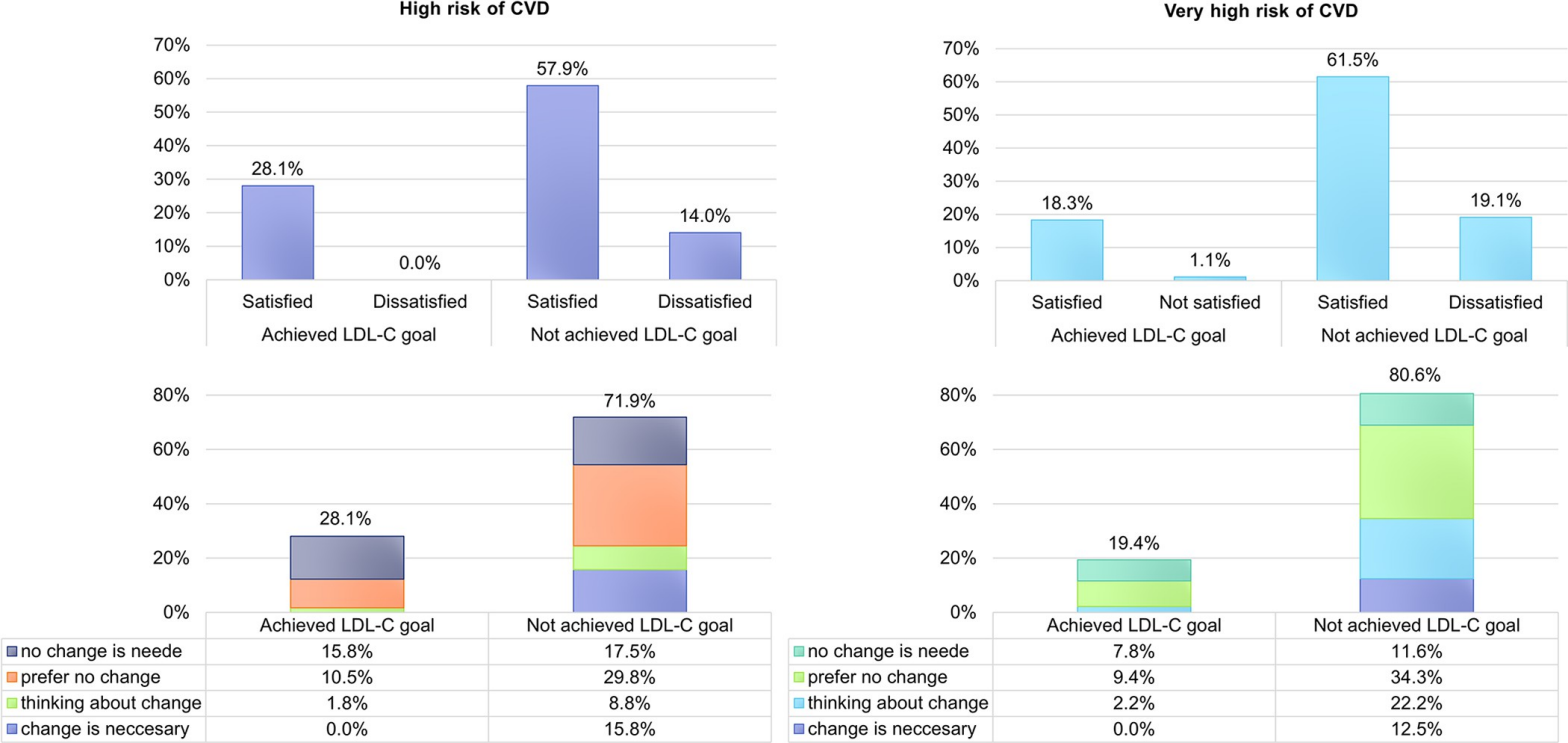
Achievement of the 2019 ESC/EAS Guidelines LDL-C goals in the context of physicians´ subjective perception of treatment outcomes in patients at high risk (A) and very high risk (B). The satisfaction was evaluated based on grouped responses to the question: *How satisfied are you with the outcome of treatment for this patient*? *(completely satisfied/rather satisfied/rather dissatisfied/completely dissatisfied)*. The physicians´ attitudes towards the change of treatment comprise from responds to the question: *Given the overall success of the patient’s treatment*, *are you considering a change in pharmacotherapy*? *(Change is necessary/ I’m thinking about the change/I lean towards the existing treatment/ There is definitely no need to change the treatment)*. The responds are shown as a percentage out of the total responds for very high and high risk patients separately.

**Fig 2:**
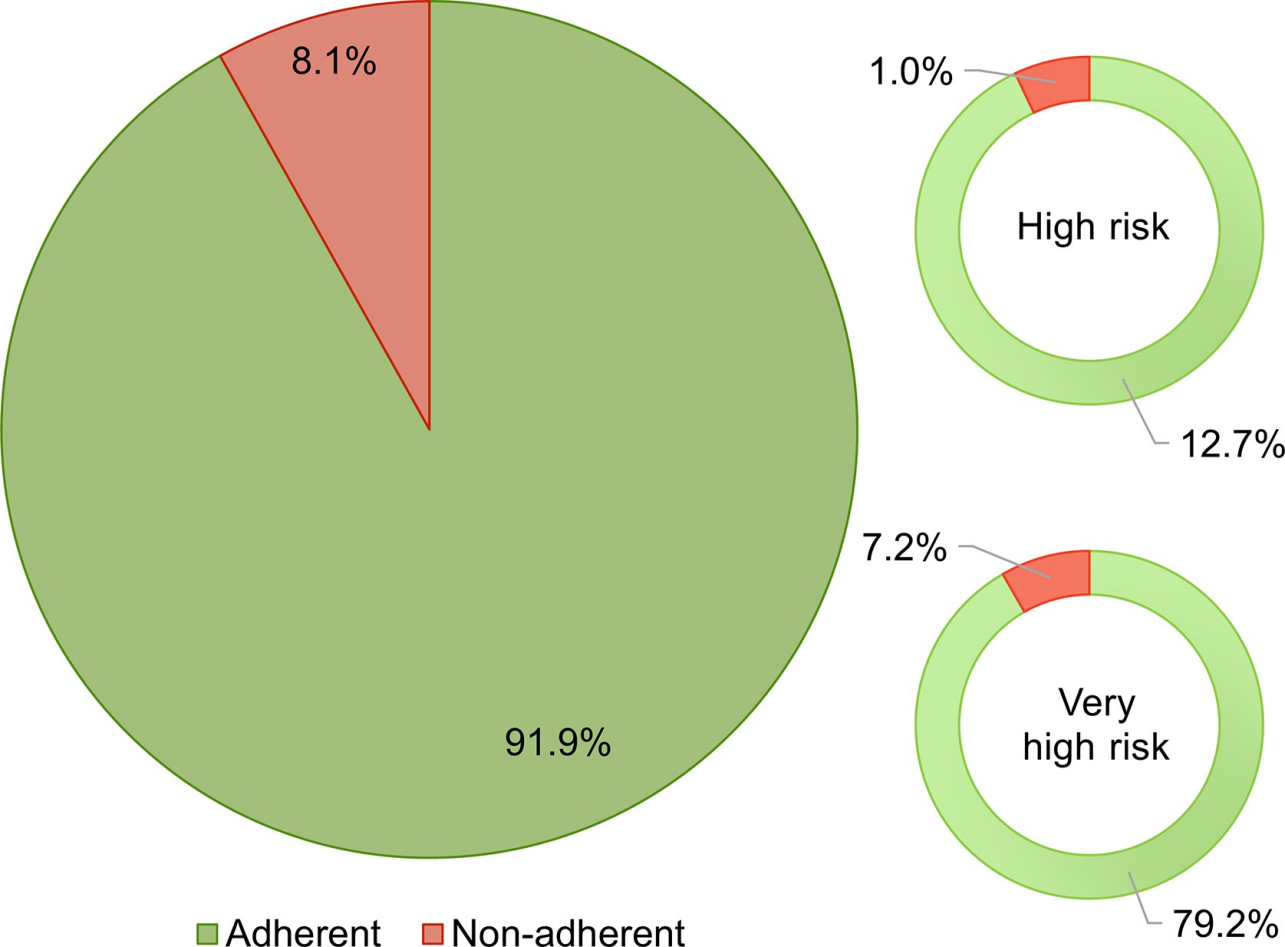
Patient adherence to pharmacotherapy. The adherence was evaluated based on grouped responds to the question: *How do you perceive the patient’s adherence to pharmacotherapy*? *(very adherent/rather adherent/rather non-adherent/very non-adherent)*. The responds are shown as a percentage out of the total responds for all analysed patients and for very high- and high risk patients separately.

Generally, only 20.5% of the patients reached the 2019 LDL-C goals– 19.4% of very high risk patients and 28.1% of high risk patients, respectively. If the less strict 2016 goals were considered, it would be 38%, and 72.4%, respectively. Despite the poor attainment of LDL-C goals, the physicians stated they were satisfied with the outcomes of the treatment in almost three quarters of patients in each risk group whose LDL-C values were above the recommended goals. According to the physicians, the change in treatment was necessary, or at least considered, in 34.6% of those very high-risk patients not achieving their goals. The percentage was even lower for those at high risk of ASCVD– 24.6%, even though 91.8% of all participating patients were recognized by their physicians as adherent to pharmacotherapy (Figs 1A, 1B and 2).

### Utilization of lipid-lowering therapy

**Fig 3:**
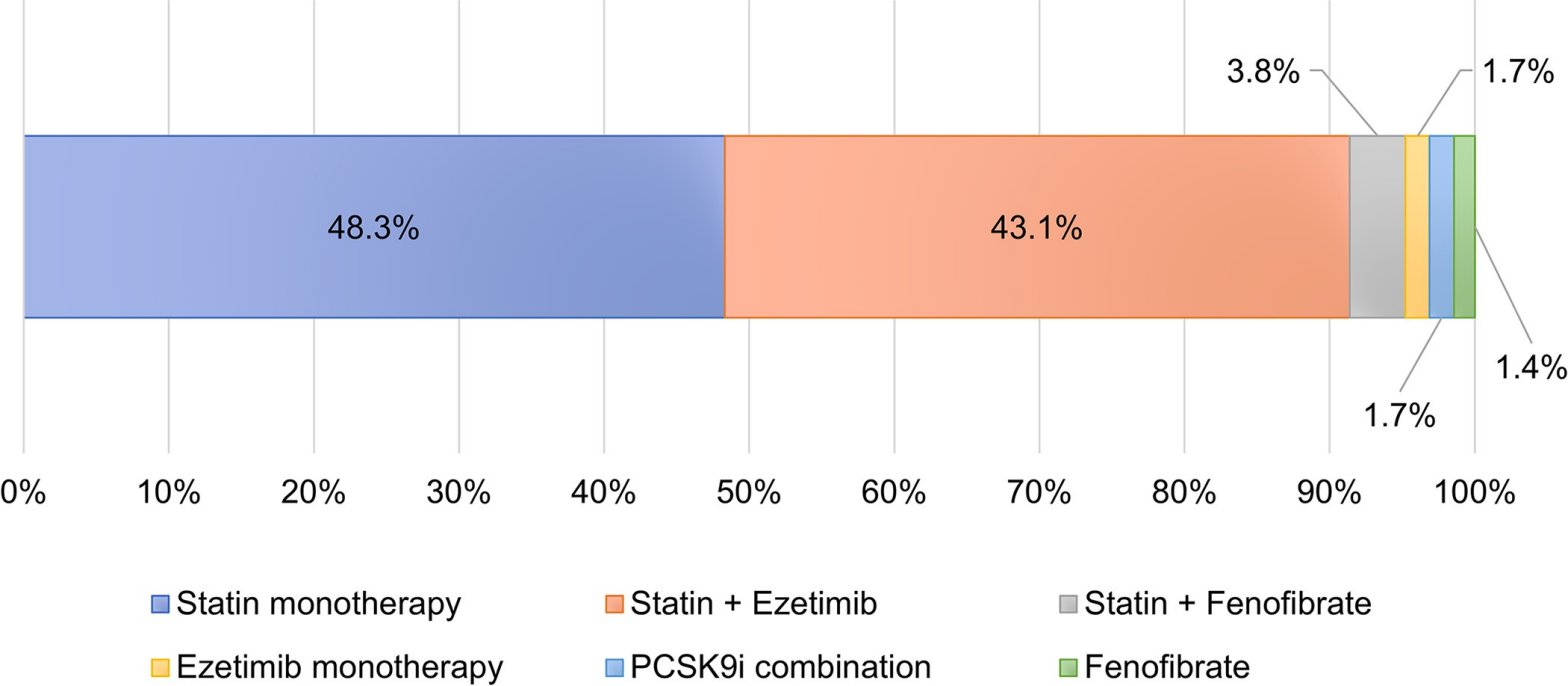
Portion of LLT types prescribed among analysed patients. In addition to LLT, 1.2% patients were prescribed with other cardiological drugs (Perindopril and/or Amlodipine).

All analysed patients were receiving lipid-lowering therapy comprising 48.3% statin monotherapy, 43.1% statins in combination with ezetimibe, 3.8% statins in combination with fenofibrate, and 4.8% of non-statin LLTs, including 7 patients receiving PCSK-9 inhibitors. High-intensity statins and statins with ezetimibe were predominately used in patient at very high risk, especially in patients with a history of a CV event (Fig 3).

**Fig 4:**
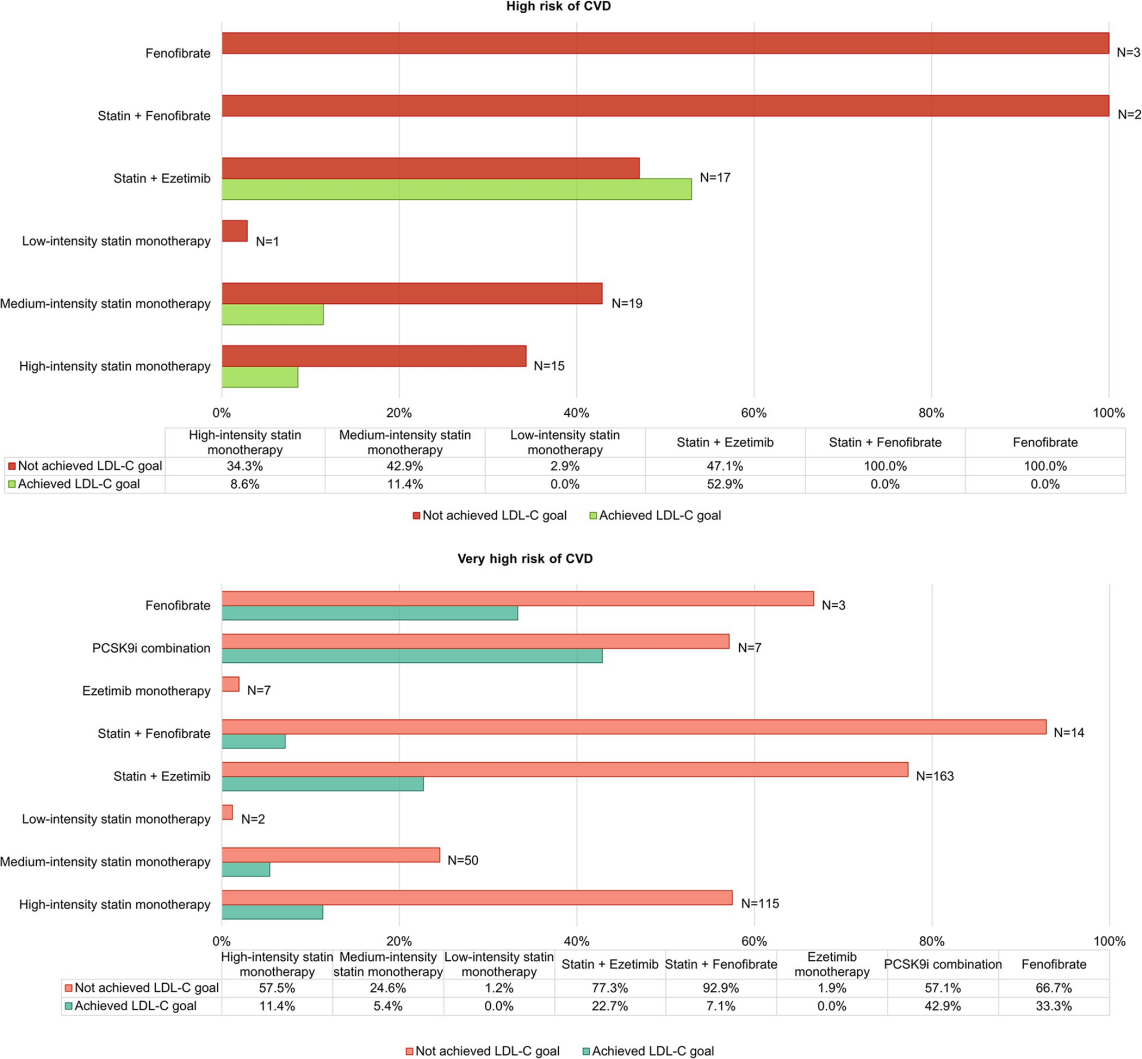
LDL-C goals attainment summarized by LLT regiments in patients at high risk (A) and very high risk (B). N is the number of patients receiving the corresponding type of LLT.

Among very high risk patients receiving statin monotherapy, the 2019 LDL-C goal attainment was 5.4% and 11.4% at moderate- and high-intensity statin therapy, respectively, and 22.7% for those receiving the combination of ezetimibe and statin. Among the high risk patients, it was 11.4% and 8.6%, respectively, and 52.9% for the statin-ezetimibe combination. 3 of 7 patients receiving PCSK9i reached the LDL-C goal (Fig 4A and 4B).

The unsatisfactory level of LDL-C (62%) and unsatisfactory level of total cholesterol (20%) were referred to as the two main reasons for initiating the current pharmacotherapeutic regimen in patients.

The theoretical therapy efficacy, e.g., the LDL-C value that could be achieved in ideal conditions by titrating the statin dosage or/and by adding ezetimibe to the regimen, was calculated for those patients who did not attain the recommended goals and were not treated with maximum statin doses/ezetimibe combination (patients with known adverse events/statin intolerance were excluded). The percentage of the maximum reduction of LDL-C was calculated as it was shown previously in randomized clinical trials for each drug/combination [14, 15]. 82 patients from 332 goal-not-achieving patients could attain the recommended LDL-C goal if their LLT had been optimized. 114 patients at very high risk of ASCVD and treated with a combination of a statin and ezetimibe would not be able to achieve the LDL-C goal, even when maximizing LLT doses, and should be considered for initiation of PCSK9i therapy (Table 2). 75% (86/114) of these patients met the indication criteria for administration of PCSK9i and considering the hypothetical maximum possible reduction of LDC-C, i.e., a 60% reduction [16, 17], all 86 patients would achieve the 2019 LDL-C goal.

**Table 2:** Therapy potential.

High risk of CVD			Current achievement of LDL-C goal		Would Achieve LDL-C goal		
** Current therapy**	**Therapeutical potential**	**Max. possible reduction of LDL-C**	**Yes**	**No**	**Yes**	**No**	**+ 28% in total**
High-intensity statin monotherapy	+ Ezetimib	-20%	3	12	7	8	
Medium-intensity statin monotherapy	High-intensity statin + Ezetimib	-26%	4	14	12	6	
Low-intensity statin monotherapy	High-intensity statin + Ezetimib	-50%	0	1	1	0	
Statin + Ezetimib	High-intensity statin	-6%	9	7	10	6	
Statin + Fenofibrate	+ Ezetimib	-20%	0	2	2	0	
**Very high risk of CVD**			**Current achievement of LDL-C goal**		**Would Achieve LDL-C goal**		
** Current therapy**	**Therapeutical potential**	**Max. possible reduction of LDL-C**	**Yes**	**No**	**Yes**	**No**	**+ 13.9% in total**
High-intensity statin monotherapy	+ Ezetimib	-20%	19	96	38	77	
Medium-intensity statin monotherapy	High-intensity statin + Ezetimib	-26%	9	41	27	23	
Low-intensity statin monotherapy	High-intensity statin + Ezetimib	-50%	0	2	0	2	
Statin + Ezetimib	High-intensity statin	-6%	37	120	43	114	
Statin + Fenofibrate	+ Ezetimib	-20%	1	13	1	13	
Ezetimib monotherapy	+ High- intensity statin	-50%	0	7	7	0	

The number of patients in both risk groups who could reach the 2019 ESC/EAS Guidelines LDL-C goals if the intensity of their LLT was maximized. The percentage of the maximum reduction of LDL-C was calculated as it was shown previously in randomized clinical trials for each drug/combination [14, 15]

### Physicians’ attitudes in the management of patient with dyslipidaemias

**Fig 5:**
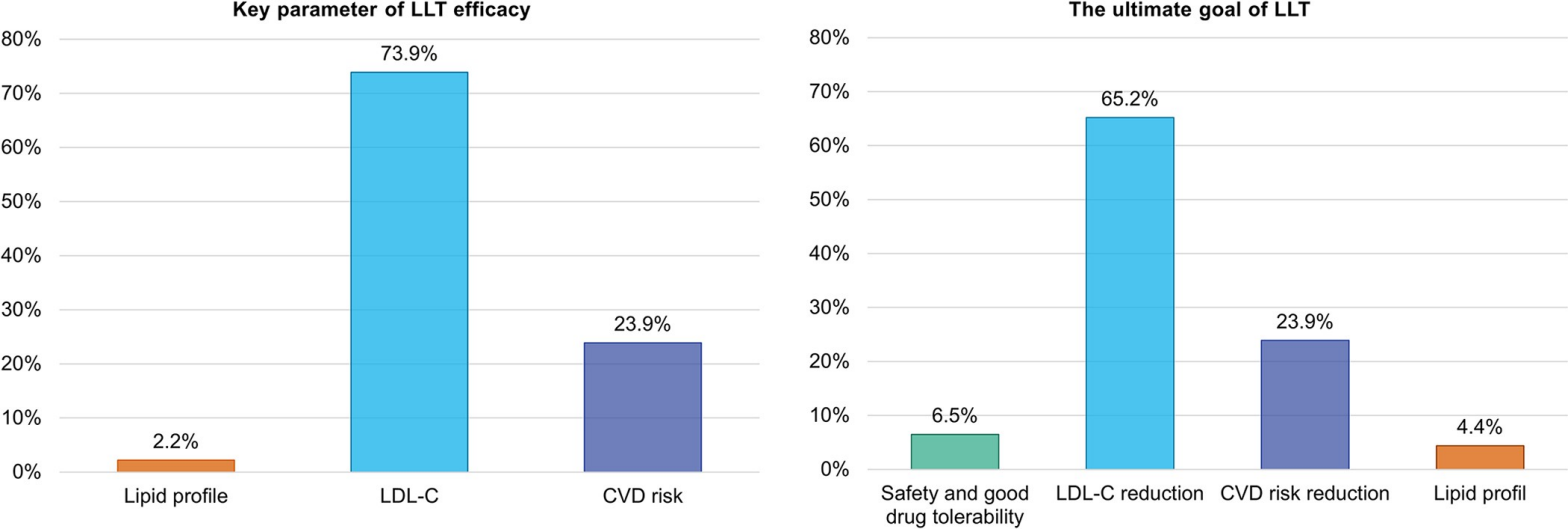
The objective target (parameter) of LLT efficacy and the ultimate goal of LLT according to physicians. The percentage represents the portion of responds to the questions: *What do you consider to be a key parameter of the LLT efficacy*? *For you*, *what is the ultimate goal of lipid-lowering therapy*? N = 46 (number of responding clinicians).

In addition to the patient records, the participated physicians completed the questionnaire mapping their personal preferences and attitudes in the treatment of dyslipidaemias. As an objective measure of LLT efficacy, 73.9% of the physicians selected LDL-C, 23.9% CV risk reduction, and 2.2% total lipid profile. LDL-C was considered as the primary target of LLT by 65.2% of physicians, while 23.9% of them stated the CV risk reduction as the main target (Fig 5).

The guidelines recommend intensive up-titration of the statin dose or initiating a combination LLT with the aim to achieve the LDL-C goals as soon as possible. However, only 17% of physicians followed those recommendations once a patient failed to reach LDL-C goals with the currently prescribed therapy. Moreover, 61% of the physicians preferred to slowly the dose. At the same time, the efficacy (83%) and the safety/tolerance (74%) were regarded as key criteria for the treatment initiation or change (S1 Fig).

**Fig 6:**
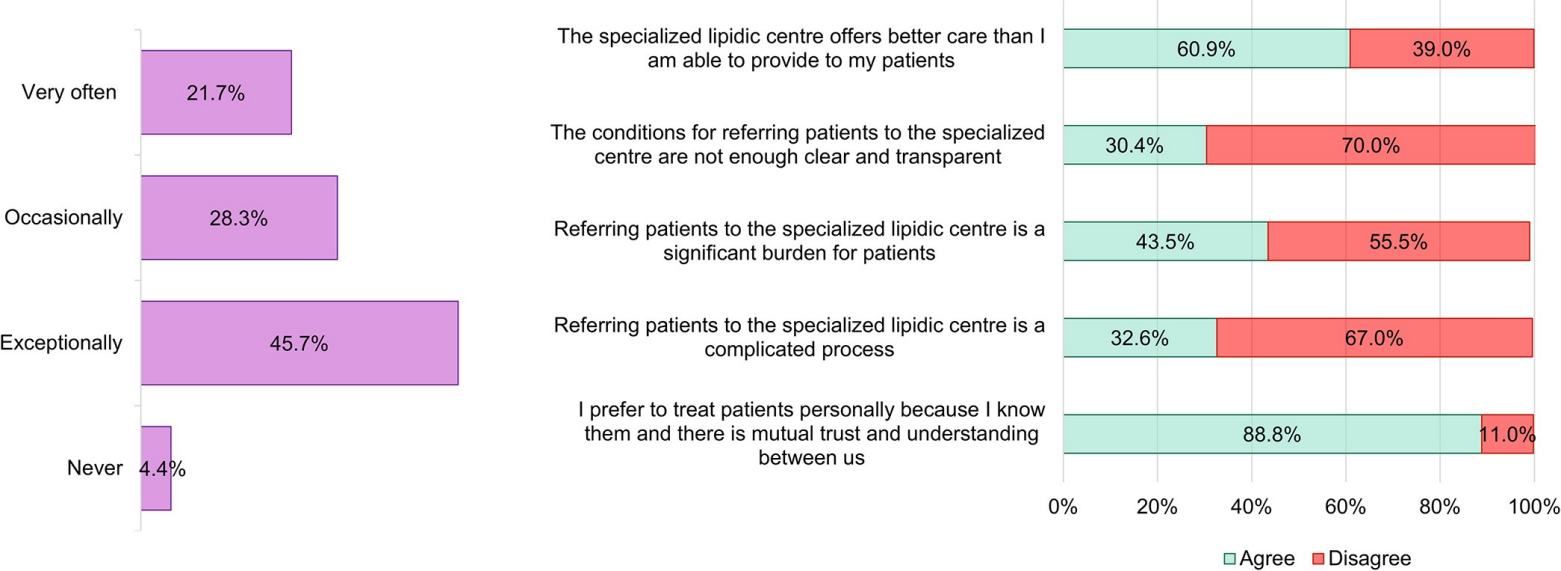
Physicians’ attitude toward the specialised lipidemic centres entitled to prescribe and administer PCSK9 inhibitors and the referral process. The percentage represents the portion of responds to the questions: A 1. *In general*, *how often do you consider referring a patient to the lipidemic centre for PCSK9 inhibitors treatment*? B *2*. *To what extent could your attitude to refer a patient to the lipidemic centre for PCSK9 inhibitors treatment be characterized by these words*? N = 46 (number of responding clinicians).

**Fig 7:**
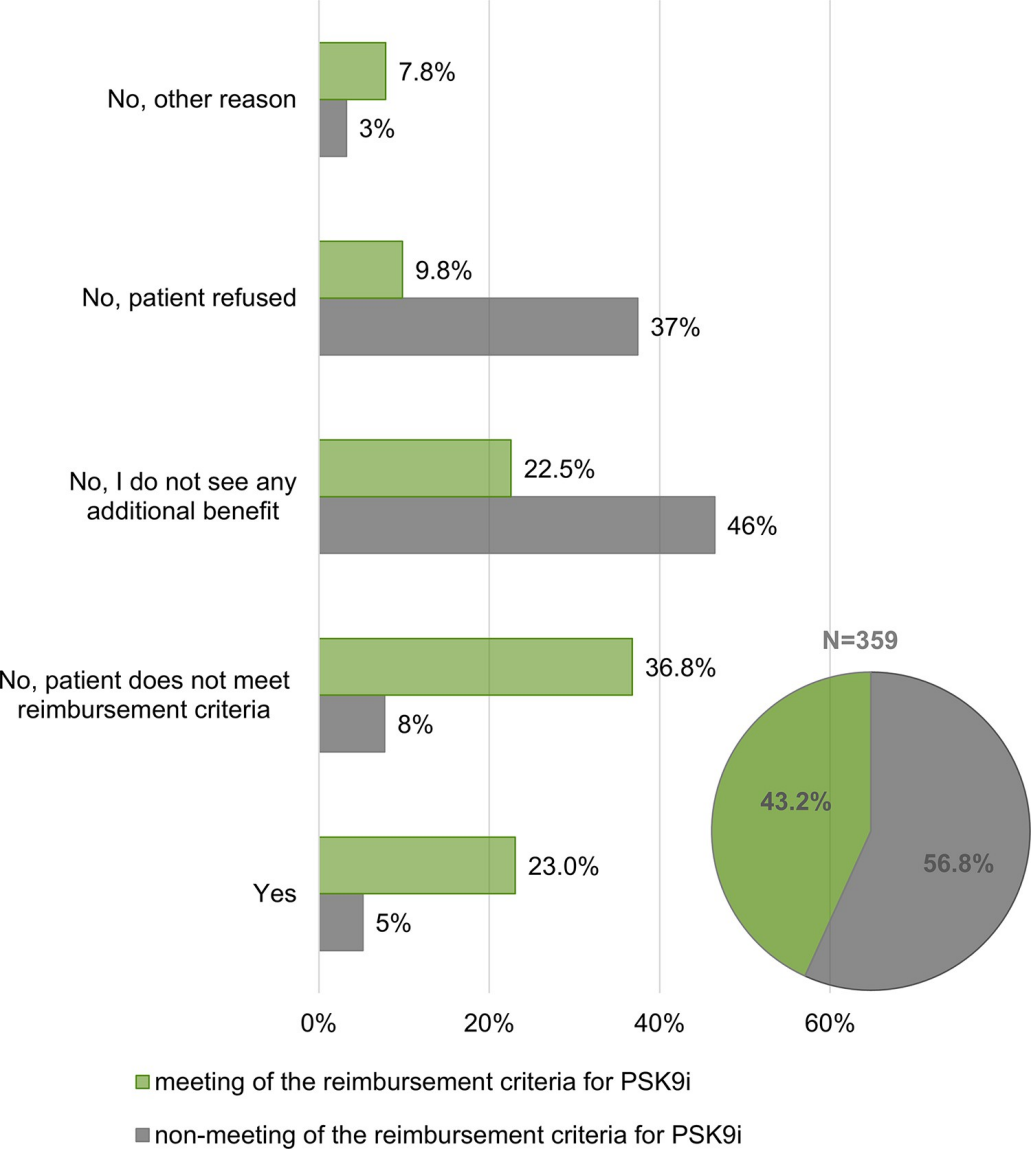
Sub-group of patients diagnosed with familial hypercholesterolemia and patients after cardiovascular event(s) stratified according to the meeting/non-meeting of the reimbursement criteria for treatment with PSK9 inhibitors. A sub-group of patients with FH and at a very high risk after CV event(s) (N = 359) were analysed and stratified according to the meeting of the official reimbursement criteria for PSK9 inhibitors treatment (Pie chart) (S1 File). The bar chart represents portion of physicians´ responds to the questions *Would you consider referring the patient to the centre for PCSK9i treatment*? for the sub-groups.

The reason for the relatively small number of enrolled patients (7 patients) taking PCSK9 inhibitors was the fact that this relatively recently introduced lipid-lowering therapy can be prescribed only in specialized centres in the Czech Republic, while the study-participating doctors were recruited from regional clinics and hospitals. Typically, local cardiologists and internists diagnose candidates for PCSK9i treatment and refer them to the centres. The data showed that although two thirds of the doctors were convinced that specialized centres may provide better care for patients, up to 74% of them recommend patients to the centre only occasionally/exceptionally and 88.8% stated that they prefer to treat patients by themselves (Fig 6A and 6B). This corresponds to the finding that in only 23% of patients who objectively met the reimbursement criteria for PCSK9i treatment based on our stratification, the physicians were considering referral to the centre, while for up 22.5% of those patients they did not state another benefit in the treatment resulting from referring to the centre (Fig 7).

## Discussion

The presented cross-sectional study conducted among selected Czech cardiologists and internists showed that, overall, 76% of patients at very high or high risk of ASCVD had not reached their risk-based LDL-C goals as defined by the guidelines. Although a declining trend can be observed when comparing the last two LDL-C measurements, the LDL-C values at enrolment to the study were still far from the desired goals. For many patients these remain unattainable due to their sub-optimal LLT regimen. The results are in line with another Czech study, the LipitenCliDec, in which 75% of primary prevention patients at very high or high risk of ASCVD displayed poorly controlled levels of LDL-C [13, 18].

The lack of adherence/compliance of the patients to the therapeutic regimen is often perceived as the major contributor to weak therapeutic outcomes in case of many chronic non-communicable diseases including dyslipidaemias. However, recent studies have shown that achieving the LDL-C goals in the groups of adherent patients is still rather poor and that reasons for this can be at least partially found on the physicians’ side [19, 20]. In the presented study, 91.9% of the enrolled patients were marked as adherent to pharmacotherapy by their attending specialists. Despite this was a subjective assessment; the high proportion of adherent patients who did not meet the required LDL-C values suggested other reasons for the observed situation than the poor treatment adherence.

The authors of the DaVinci study mentioned, within grounds of failure to achieve the 2019 ESC/EAS guideline LDL-C goals, the possibly poor penetration of new recommendations among the clinicians [9]. Therefore, one of the aims of this study was to evaluate the physicians’ preferences for the treatment of dyslipidaemias and the management of patients at a very high/high risk of ASCVD in the context of the existing guidelines. From the physicians´ mixed responses to questions what they consider to be the objective and goal value for the lipid-lowering treatment, it seems that the ultimate objectives and goals clearly defined by the guidelines are still not widely recognized by the clinicians. Incomplete knowledge of the guidelines is also indicated by the fact that in 20% of all patients enrolled, physicians incorrectly assessed the patient’s ASCVD risk category.

Surprisingly, for up to 61.5% of patients at very high risk of ASCVD who did not meet the LDL-C goals, the physicians recorded subjective satisfaction with the treatment outcomes and did not consider any regimen change. This is even more surprising given the fact that most of these patients have not reached maximum possible intensity of treatment and that greater reduction of LDL-C could have been achieved by increasing the intensity of statin therapy and/or adding ezetimibe to the regimen. If this happened in all the patients who did not experience any statin/ezetimib intolerance, the percentage of very high risk patients who would have achieved the LDL-C goals would increase from 19.4% to 33.2%, respectively from 28.1% to 56.1% in patients at high risk. Bearing in mind the well-known LDL-C principle, e.g. that each 1 mmol/L reduction in LDL-C reached by statins therapy reduce major vascular events by 22% [21] underlines the need to strive for the maximum possible reduction in LDL-C by maximizing the statins treatment.

The patients in whom LDL-C levels cannot be reduced to goal values with maximum statin dose in combination with ezetimibe, and particularly for those with the established ASASCVD, PCSK9 inhibitors should be considered. In the Czech Republic, those patients need to be referred to a specialized centre where PCSK9i can only be prescribed under rather restrictive reimbursement criteria. Our data indicate that there is generally low willingness to modify the therapeutic approach of a patient by referral to the centre. Most frequent reason for this is the personal preferences of physicians to treat the patient personally and, on the other hand, the complexity of referral process or its conditions. We identified 204 patients, 34 with FH and 170 after a CV event, who objectively met the current reimbursement criteria (S1 File) and could benefit from the PCSK9 treatment. However, physicians considered referral of only one-fifth of those patients to the centre. The main reason for not referring was the inaccurate evaluation of the patients from the perspective of reimbursement conditions. The second most frequently occurring reason of no additional benefit of PCSK9 inhibitors in these patients seems particularly surprising. Although PCSK9 inhibitors are widely known within the clinicians´ community, further education seems to be needed regarding the enormous benefits for patients at very high risk of ASCVD with dyslipidaemia.

There are few limitations of this study that must be acknowledged. It is a cross-sectional survey so a cause-and-effect relationship cannot be established. However, our results indicate suboptimal utilization of LLTs and lack of willingness to use combinations as causes of sub-optimal control of blood lipid levels. However, further studies are needed to assess causality. All data were reported by physicians, so bias due to self-reporting cannot be excluded. As the patient compliance was subjectively assessed by specialists, bias in assessing the correct dose-response may have occurred and higher than necessary dose may have been prescribed. If a proportion of these patients were more consistently compliant with treatment, they would achieve lower plasma LDL-C concentrations and hence the total number of patients achieving the proposed target would be higher. The blood level of LDL-C before the first administration of LLT was not available to calculate the recommended 50% reduction from baseline and therefore only the actual LDL-C values were considered.

In conclusion, the presented study showed that among very high and high-risk patients receiving lipid-lowering therapy, with relatively high treatment adherence, the LDL-C goals attainment is very low and LLT utilization is sub-optimal in a large group of patients. The analysis of physicians´ attitudes toward dyslipidaemia management of patients at risk together with patient chart review indicate non-optimal compliance of clinicians to the guidelines in a real-world setting. However, further research is required for validation, addressing the demand to increase the intensity of therapy with an emphasis on active lowering of blood cholesterol seems like a good starting point for the gradual elimination of the enormous unmet need that the unsatisfactory LDL-C values represent. The total cost of ASCVD in the EU is up to 210€ billion comprising of 111€ billion of health care costs, 54€ billion of productivity losses and 45€ billion due to indispensable care of people with ASCVD (EHN, 2017). The uncontrolled LDL-C blood level as a well-established risk factor of ASCVD thus represents not only a medical problem, but also a huge economic burden on national health and social systems. It is therefore essential to pay attention to lowering LDL-C and other risk factors in the prevention of ASCVD in populations despite some intrinsic problems such as a suboptimal adherence. There is still unexploited potential in conventional lipid-lowering therapy in the Czech Republic and probably elsewhere in Europe. As for improving adherence as the most promising and free intervention, physicians could survey their patients’ adherence using information systems such as the electronic prescription and claims databases available to them. For example, calculating medication possession ratios offers a tool to assess patient medication availability and thus alert physicians to suboptimal adherence [22]. Healthcare payers could communicate and create incentives to motivate physicians and patients to better understand and achieve LDL-C targets. Physicians could be incentivized to monitor LDL-C by better understanding and more closely linking its target values to the prescribed dose of medication, while patients could be provided with a planner for their visits to ensure timely access to medication. Given the findings herein, patients and payers would, with a minimum use of additional resources, instantly benefit from a tighter focus on the LDL-C.

## Supporting information

S1 Table The LDL-C blood values and the average time between the follow-up cholesterol checks. SD—standard deviation.
(DOCX)

S1 Fig Physicians attitudes in case of insufficient patient response to the lipid-lowering therapy. The percentage represents the portion of responds to the questions: 1. *Which of the following statements best describes your attitude in case of insufficient response to treatment with lipid-lowering drugs*? N = 46.
(DOCX)

S1 File The current reimbursement criteria for PCSK9 inhibitors in Czech Republic (17.5.2022). (DOCX)
