# Supplementary material for: Low LDL-C goal attainment in patients at very high cardiovascular risk due to lacking observance of the guidelines on dyslipidaemias
Source: PLoS One. 2023 May 22;18(5):e0272883. doi: 10.1371/journal.pone.0272883 (PMC10202298; doi:10.1371/journal.pone.0272883)
Supplement: S1 Table — SD—standard deviation. (DOCX) [file pone.0272883.s001.docx]

# **Supporting information S1**

**Table: The LDL-C blood values and the average time between the follow-up cholesterol checks.** SD - standard deviation.

| **Risk category** | **Very high + High** | **Very high** | **High** |
| --- | --- | --- | --- |
| **Actual LDL-C (mmol/L) mean (SD)** | 2.21 (1.00) | 2.18 (0.96) | 2.36 (1.21) |
| **Previous LDL-C (mmol/L) mean (SD)** | 2.55 (1.23) | 2.53 (1.24) | 2.69 (1.14) |
| **Change in LDL-C (mmol/L) mean (SD)** | -0.29 (1.11) | -0.28 (1.02) | -0.32 (1.54) |

|  | **less than 12 months** | **12-18 months** | **more than 18 months** |
| --- | --- | --- | --- |
| **Follow-up LDL-C measurement** | 85.2% | 9.1% | 5.7% |
