# Supplementary material for: Low LDL-C goal attainment in patients at very high cardiovascular risk due to lacking observance of the guidelines on dyslipidaemias
Source: PLoS One. 2023 May 22;18(5):e0272883. doi: 10.1371/journal.pone.0272883 (PMC10202298; doi:10.1371/journal.pone.0272883)

# **Supporting information S2**

**Figure:** **Physicians attitudes in case of insufficient patient response to the lipid-lowering therapy**. The percentage represents the portion of responds to the questions: 1*. Which of the following statements best describes your attitude in case of insufficient response to treatment with lipid-lowering drugs?* N=46


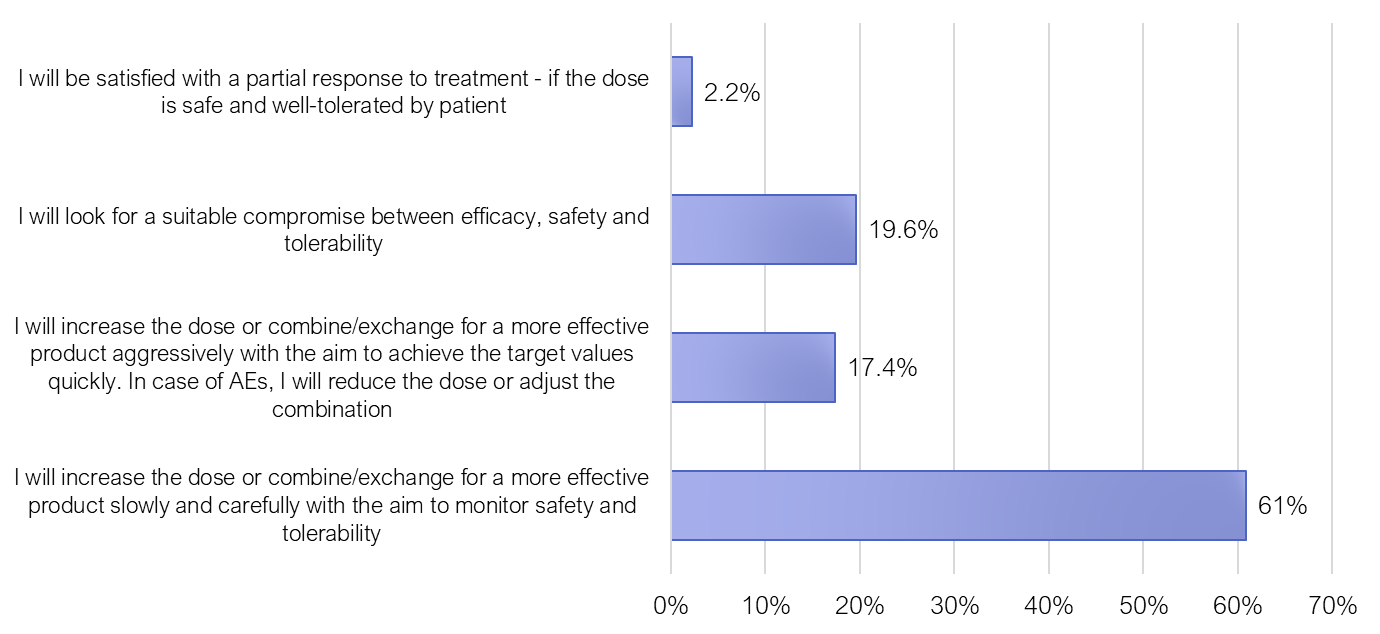

Supplement: S1 Fig — The percentage represents the portion of responds to the questions: 1. Which of the following statements best describes your attitude in case of insufficient response to treatment with lipid-lowering drugs? N = 46. (DOCX) [file pone.0272883.s002.docx]
