# Supplementary material for: Low LDL-C goal attainment in patients at very high cardiovascular risk due to lacking observance of the guidelines on dyslipidaemias
Source: PLoS One. 2023 May 22;18(5):e0272883. doi: 10.1371/journal.pone.0272883 (PMC10202298; doi:10.1371/journal.pone.0272883)
Supplement: S1 File — (DOCX) [file pone.0272883.s003.docx]

# **Supporting information S3**

**The current reimbursement criteria for PCSK9 inhibitors in Czech Republic (17.5.2022).** Alirocumab or evolocumab is reimbursed in patients adhering to dietary measures and existing hypolipidemic therapy: 1) with heterozygous familial hypercholesterolaemia, or 2) with non-familial hypercholesterolaemia or mixed dyslipidemia at a very high cardiovascular risk with a manifesting atherosclerotic disease, for which their current high-intensity hypolipidemic treatment was not effective enough to achieve LDL-C levels of at least 3.1 mmol/l in the case of heterozygous familial hypercholesterolaemia without a developed cardiovascular disease, or at least 2.5 mmol in patients at a very high risk in secondary prevention. These LDL-C criteria also apply to reimbursement in patients for whom statin treatment is demonstrably contraindicated or intolerant. High-intensity hypolipidemic therapy is defined as a therapy with the maximum tolerated dose of atorvastatin or rosuvastatin, in the case of proven intolerance to both, the maximum tolerated dose of any statin, in combination with another hypolipidemic agent such as ezetimibe, in the case of statin intolerance by the hypolipidemic drug alone, if indicated. The non-use of ezetimibe in existing hypolipidemic therapy must be medically justified in the patients' medical records. Statin intolerance is defined as the intolerance of at least two consecutive statins that leads to their discontinuation. The intolerance of both statins must then be demonstrated as a decrease in clinical symptoms or normalization of CK after discontinuation and recurrence of myalgia or increase in CK after the re-introduction of statin. CK elevations not exceeding 4 times the upper limits without clinical symptoms cannot be considered as statin intolerance. The effect of alirocoumab or evolocumab therapy is regularly evaluated; first recorded in the clinical documentation no later than 12 weeks after the start of treatment. Reimbursement of alirocoumab or evolocumab is terminated in the case of demonstrable non-cooperation of the patient or ineffectiveness of the treatment consisting in failure to reach the target values of LDL-C in the 24th week of therapy. 1 subcutaneous injection of 75 mg once every 14 days is covered from the public health insurance funds.
